# Familial Deep Vein Thrombosis in a Child With Antithrombin III Deficiency: A Case Report

**DOI:** 10.7759/cureus.54157

**Published:** 2024-02-13

**Authors:** Shivani Kale, Devika Jadhav, Sampada Tambolkar, Avinash Daru

**Affiliations:** 1 Pediatrics, Dr. D. Y. Patil Medical College, Hospital & Research Centre, Dr. D. Y. Patil Vidyapeeth, Pune, Pune, IND

**Keywords:** lower-molecular-weight heparin, rare genetic diseases, familial disease, deep vein thrombosis (dvt), thrombosis

## Abstract

Deep vein thrombosis (DVT) is caused by a clot (thrombus) formed in the deep veins, usually the legs. The incidence of DVT is notably less prevalent in children than in adults. Here, we present a rare case of DVT in an eight-year-old female child with a significant family history involving the untimely death of the maternal aunt. The child presented with pain and edema in the left lower limb causing immobilization without any obvious cause. The clinical features suggested the possibility of DVT. On further evaluation and radiological investigations, the diagnosis of DVT was confirmed. A complete thrombophilia workup was done showing antithrombin (AT) III deficiency. The patient was then started on low-molecular-weight heparin, leading to improvement in the symptoms. Oral rivaroxaban was continued for the patient on discharge.

## Introduction

Deep vein thrombosis (DVT) is characterized by the formation of blood clots (thrombi) within the deep veins, typically in the lower limbs. These clots also have a tendency to detach from their origin and migrate through the bloodstream, ultimately reaching the lungs and causing pulmonary embolism (PE) [1]. DVT is a manifestation of venous thromboembolism. Venous thromboembolism is described by Virchow’s triad: endothelial injury, stasis, and hypercoagulability [2]. The incidence of DVT in the general population is estimated to be 5/10,000 hospital admissions or 0.07/10,000 in children annually [3].

The presence of a central venous catheter is the most common risk factor for venous thromboembolism in the pediatric age group, followed by inherited hypercoagulable states, infection, immobility, trauma, malignancy, genetic predispositions, and chronic inflammatory conditions [4]. DVT is a critical medical concern as it can lead to significant morbidity and mortality if not diagnosed and treated promptly.

Antithrombin (AT) previously known as AT3 acts as an inhibitor for thrombin (factor 2a) and factor 10a in the coagulation pathway. Deficiency in AT can arise from either inherited or acquired causes. Inherited deficiencies are attributed to mutations in the AT gene [5]. Acquired deficiencies can result from impaired production of functional AT, such as in cases of liver disease, protein losses like those observed in nephrotic syndrome, and accelerated consumption as observed in disseminated intravascular coagulation (DIC) [6]. Inheritance of AT deficiency is autosomal dominant with prevalence found to be around 0.02-0.2%, seen equally common in males and females.

This case report aims at emphasizing the clinical significance, familial nature of the etiology, and diagnosis of this vascular disorder.

## Case presentation

An eight-year-old female belonging to the Kuli Maratha clan presented to the outpatient department (OPD) with chief complaints of swelling and pain in the left leg for six days. The patient initially developed left flank pain progressing to the left lower limb with swelling and edema. On taking a brief medical history, the patient had no complaints of chest pain, breathlessness, and dizziness. No history of trauma or prolonged immobilization was reported.

Upon taking a family medical history, the patient was born out of a third-degree consanguineous marriage, second in birth order with a history of DVT in the maternal grandfather, maternal uncle, and maternal aunt, leading to two episodes of pulmonary thromboembolism in the maternal uncle followed by a medial malleolar ulcer. The maternal aunt had a thromboembolic episode leading to pulmonary embolism causing death in the postpartum duration.

On examination, the patient was stable with a pulse rate of 86/min, respiratory rate 26/min, BP 110/60 mmhg, and Spo_2_ 98% on room air.

**Figure 1:**
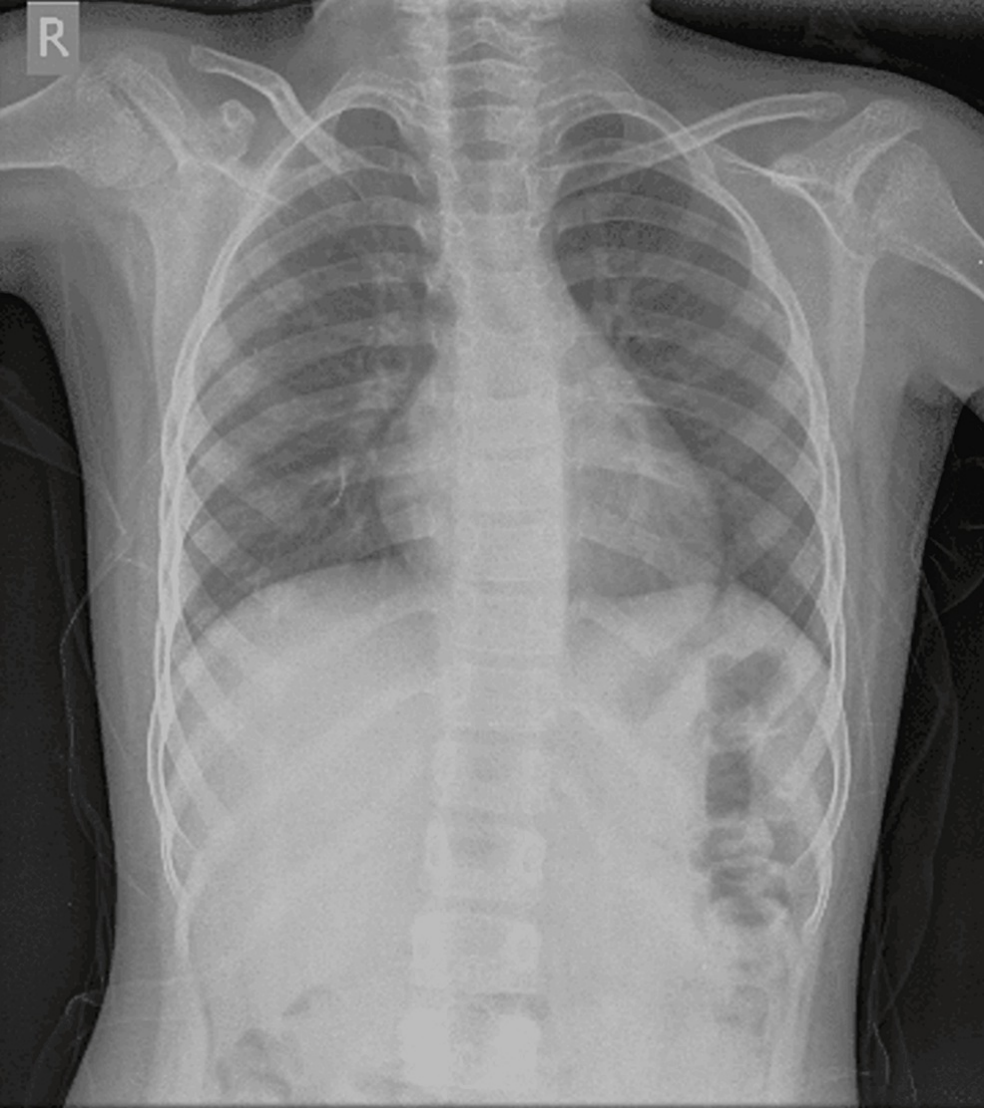
Chest radiograph - posteroanterior view

Local examination revealed erythema, warmth, swelling, and tenderness localized to the left lower limb. Peripheral pulses, namely, the femoral and posterior tibial, were well felt. Homan’s sign was found to be positive in the left lower limb, described by pain in the calf on forced dorsiflexion of the ankle. On palpation, the abdomen was soft and non-tender. On auscultation, the chest was clear with bilaterally equal breath sounds (Figure 1), and no cardiac murmur was heard. Laboratory investigations were done, and the profile is shown in Table 1. Table 2 shows the arterial blood gas results.

**Table 1:** Laboratory investigations

Hemogram	Result	Reference interval
Hemoglobin	9.9	12.0–14.5 g/dl
Total leucocyte count	11,300	4000–10800/ul
Neutrophils	61%	
Lymphocytes	31%	
Platelet count	379,000	150000–410000/ul
Red blood cell count	3.47	4.10–5.30 x 10^6^/ul
Hematocrit	29.50	35.7–43.0%
MCV (Mean corpuscular volume)	85	78.5–90.4fL
MCH (Mean corpuscular hemoglobin)	28.4	25.0–33.0 pgms
MPV (Mean platelet volume)	6.3	7.9–10.8 fL
Liver function test		
Total biliburin	0.24	0.22–1.20 mg/dl
Conjugated bilirubin	0.11	Up to 0.5 mg/dl
Unconjugated bilirubin	0.13	0.1–1.0 mg/dl
SGOT (serum glutamic oxaloacetic transaminase)	15	8–50 U/Lt
SGPT (serum glutamate pyruvate transaminase)	08	7–45 U/Lt
ALP (alkaline phosphatase)	97	142–335 U/Lt
CRP (C-reactive protein)	116.00	>10.0 mg/L – Acute Inflammation
Renal function test		
Urea	18	17–49 mg/dl
Creatinine	0.35	0.26–0.61 mg/dL
Coagulation profile		
aPTT (activated partial thromboplastin time)	31.40	25.24–30.67 secs
PT (prothrombin time)	12.8	10.66–14.53 secs
INR (international normalized ratio) value	1.05	0.85–1.15
Vitamin B12	97.0	187–883 pg/mL
Homocysteine levels	13.39	5.08–15.39 umol/Lt
D-dimer	4,876	0–500 ng/ml

**Table 2:** Arterial blood gas

Arterial blood gas	Results	Reference range
pH	7.36	7.32–7.42
pCO_2_	44 mmhg	41–51 mmhg
pO_2_	37 mmhg	25–40 mmhg
Lactate	0.5 mEq/L	<1 mmol/l
HCO_3 _(BICARB)	24.3 mEq/L	21–28 mEq/L
Base excess	-0.5 mEq/L	
SO_2_	99%	

The left lower limb Doppler showed a left great saphenous vein (GSV) until the distal thigh region, which appeared non-compressible and showed no flow on color Doppler, suggestive of thrombosis. The external iliac vein, common femoral vein (CFV), visualized portion of the proximal DFV (deep femoral vein), and superficial femoral vein (SFV) in the thigh region appeared non-compressible and showed no flow on the color Doppler.

**Figure 2:**
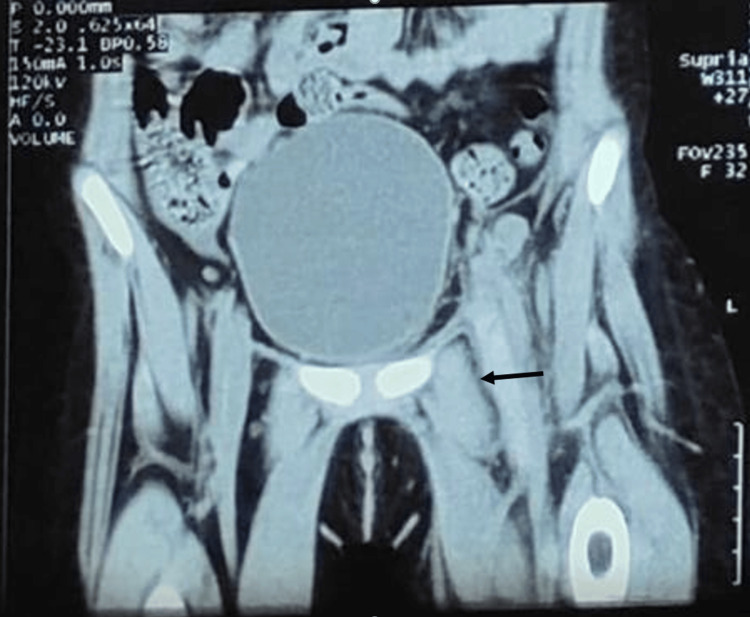
Coronal section CT scan Distended and hyperdense common external iliac, and femoral veins, suggesting thrombosis.

Furthermore, CT scan of the lower limbs was done to confirm the diagnosis, which revealed distended and hyperdense common, external iliac, and femoral veins, suggesting thrombosis and showed no evidence of any bony fracture (Figure 2).

**Figure 3:**
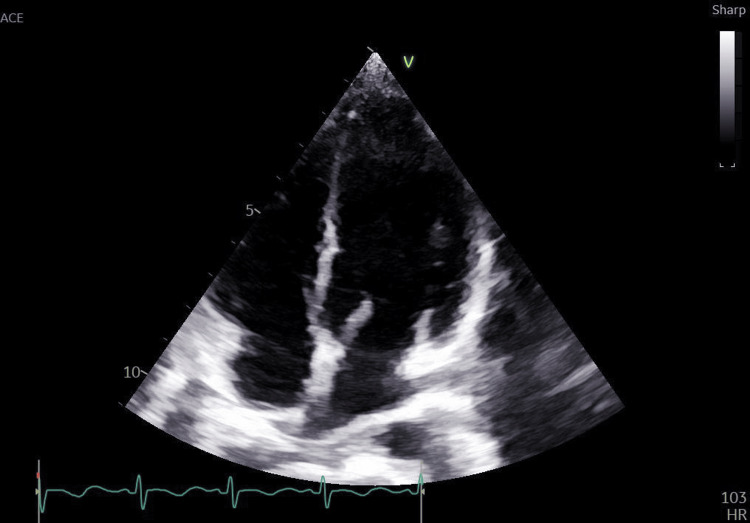
2D echo (apical four-chamber view, suggestive of normal chamber dimensions)

**Figure 4:**
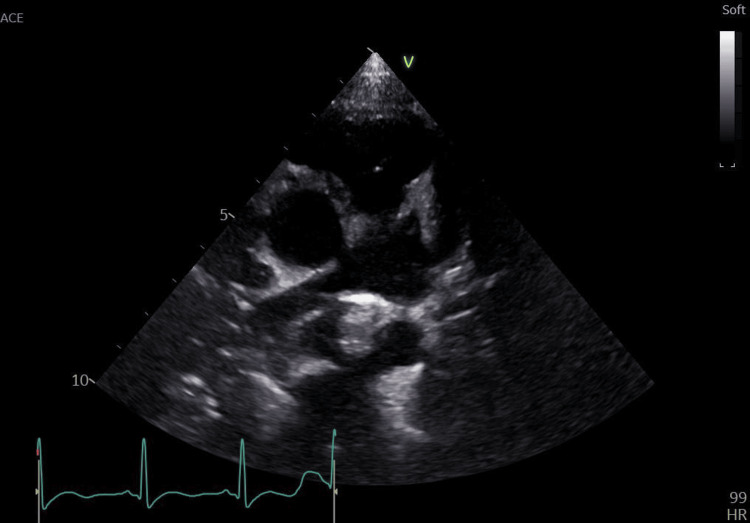
2D echo image (modified short-axis view at the aorto-pulmonary level, suggestive of a normal main pulmonary artery with branches)

2D echo showed a normal heart study and showed no right ventricular strain pattern (Figures 3, 4).

A complete thrombophilia workup was done to look for any inherited cause of DVT, which showed antithrombin III deficiency (patient's result 11.00%, normal 75-125%).

The patient was initially started on low-molecular-weight heparin subcutaneous injection at 1 mg/kg/day twice daily dosing leading to improvement in the patient's symptoms, which was then stopped after seven days, and rivaroxaban 10 mg once daily dosing was started for the patient on discharge and is continued.

## Discussion

The incidence of DVT is rare in the pediatric population with 0.07 per 10,000 hospital admissions [3]. The majority of cases reported in pediatric patients are often associated with the presence of a central venous catheter, malignancy, immobility, infection, and hereditary hypercoagulable states. Choi et al. showed that out of 25 pediatric patients, 80% had underlying primary diseases or risk factors, such as venous catheterization (24%), malignancy (20%), and systemic disease (12%), showing a relatively high incidence of malignancy [7]. In our case, the patient had complaints of unilateral leg pain and swelling without the presence of any obvious risk factors and a significant family history. Laboratory investigations showed elevated D-dimer levels with findings suggestive of DVT on CT scan and venous Doppler of the lower limbs. A full thrombophilia workup showing AT III deficiency was done for the patient due to the presence of a strong family history involving the death of the maternal aunt in the postpartum duration. Pulmonary embolism was ruled out due to the absence of any clinical features of dyspnea, tachycardia, and chest pain with stable vital parameters, with a normal chest X-ray and arterial blood gas. Furthermore, a 2D echo was done for the patient, showing no right ventricular strain pattern.

The use of parenteral anticoagulation followed by warfarin or a direct oral anticoagulant like rivaroxaban and apixaban is the mainstay of treatment [8]. The Einstein program that includes three clinical trials showed that the use of rivaroxaban was equally effective in the treatment of DVT and its complications as compared to the vitamin K antagonists [9].

In our case, the patient was treated with a low-molecular-weight heparin subcutaneous route at 1 mg/kg/day twice daily dosing for seven days and then discharged on oral rivaroxaban 10 mg single dose daily. The use of warfarin is challenging because of its narrow therapeutic range and various food and drug interactions, all of which need periodic monitoring of coagulation, dose adjustments, monitoring of interactions with other medications, and dietary limitations. Rivaroxaban is a direct-acting oral anticoagulant, having a wide therapeutic window and requiring no continuous monitoring of coagulation and no dietary limitations [9].

## Conclusions

Our case demonstrates the occurrence of DVT in a child with a significant family history and the absence of other common risk factors for thrombosis. Early anticipation, timely diagnostic workup, and early anticoagulation therapy helped in the prevention of serious life-threatening thromboembolic events. Family screening for such inherited causes of DVT would help in the early diagnosis of high-risk individuals and prophylactic anticoagulation therapy can be planned to prevent thromboembolic complications. Prenatal and antenatal counseling can also be offered. Regular follow-up, compliance with anti-coagulation therapy, and whole genome sequencing are advised.

## References

[1] Goldhaber SZ, Bounameaux H (2012). Pulmonary embolism and deep vein thrombosis. Lancet.

[2] Bagot CN, Arya R (2008). Virchow and his triad: a question of attribution. Br J Haematol.

[3] Andrew M, David M, Adams M, Ali K (1994). Venous thromboembolic complications (VTE) in children: first analyses of the Canadian Registry of VTE. Blood.

[4] Jinks S, Arana A (2019). Venous thromboembolism in paediatrics. BJA Educ.

[5] Patnaik MM, Moll S (2008). Inherited antithrombin deficiency: a review. Haemophilia.

[6] Maclean PS, Tait RC (2007). Hereditary and acquired antithrombin deficiency: epidemiology, pathogenesis and treatment options. Drugs.

[7] Choi HS, Choi CW, Kim HM, Park HW (2016). Venous thromboembolism in pediatric patients: a single institution experience in Korea. Blood Res.

[8] Hylek EM, Go AS, Chang Y, Jensvold NG, Henault LE, Selby JV, Singer DE (2003). Effect of intensity of oral anticoagulation on stroke severity and mortality in atrial fibrillation. N Engl J Med.

[9] Bauersachs R, Berkowitz SD, Brenner B (2010). Oral rivaroxaban for symptomatic venous thromboembolism. N Engl J Med.

